# Review of *Land, Investment & Politics: Reconfiguring East Africa’s Pastoral Drylands*. Edited by Jeremy Lind, Doris Okenwa & Ian Scoones. Woodbridge, Suffolk: James Currey, an imprint of Boydell and Brewer, 2020.

**DOI:** 10.1186/s13570-021-00211-7

**Published:** 2021-10-22

**Authors:** John G. Galaty

**Affiliations:** McGill University, 855 Sherbrooke St. West, Montreal, QC H3A 2T7 Canada

**Keywords:** East Africa, Drylands, Rangelands, Pastoralism, Land rights, Global financial investments, Development projects, Renewable and non-renewable energy resources, Sedentarization, Inequality

## Abstract

Edited by Jeremy Lind, Doris Okenwa & Ian Scoones

*Land, Investment & Politics: Reconfiguring East Africa’s Pastoral Drylands.*

Woodbridge, Suffolk: James Currey, an imprint of Boydell and Brewer, 2020.

224 pages, ISBN 978-1-84701-252-4 (James Currey hardback) and ISBN 978-1-84701-249-4 (James Currey paper)

Jeremy Lind, Research Fellow, Institute of Development Studies, University of Sussex. Co-editor of “Pastoralism and Development in Africa” (2013)

Doris Okenwa, Ph.D student in Anthropology, London School of Economics. Ph.D research on oil discoveries in Turkana County, Kenya.

Ian Scoones, Professorial Fellow, Institute of Development Studies, University of Sussex. Co-Director of ESRC STEPS (Social, Technological and Environmental Pathways to Sustainability) Centre, and leader of the European Research council project PASTRES (Pastoralism, Uncertainty and Resilience). Author of “Africa’s Land Rush: Rural Livelihoods and Agrarian Change”.

Eastern Africa has become the next global frontier for financial investment. Buoyed by neo-liberal legitimation, multi-national companies and international organizations are seeking to ‘develop’ (or ‘exploit’, depending on your ideological predilections) resources that up to now have been ‘undiscovered’ (a familiar trope for the continent) or at least un-available. States eager for new sources of revenue have invited the exploration and exploitation of sources of energy (oil, geothermal, solar, wind power), of cultivable land and of rangelands rich in wildlife across enormous swaths of arid and semi-arid lands. Let us ask ourselves whether this is for the good, with investments finally flowing to lands long ignored by repositories of capital, or an unfortunate resurgence of neo-colonialism, through the indirect capture of resources by global bodies seeking, on the one hand, profits, or, on the other hand, absolution, by virtue of bringing the greatest benefits to the most people. Nor is it clear that we could even identify the difference when renewable energy, increases in food production and conserving land and wildlife are concerned, which address some of the major challenges of our age in attempts to achieve a sustainable future.

Not many decades ago, scholars sought to understand the institutional dynamics of small-scale herd-owners, representatives of pastoralism, which is a form of agrarian production that arose some 6000 years ago to sustain people living in arduous drylands that may constitute half of the land mass of the earth. With appropriate alertness to issues that really matter to the inhabitants of the drylands, researchers, policy-makers and the public have increasingly tried to understand the larger-order state, regional and global contexts that frame what is crucial for local land users: expanding networks of markets that draw in livestock and circulate local and imported goods, the proliferation of village schools, clinics, NGO’s government offices, and the diversification of rural economies, from Tibet, Mongolia, central Asia, the Middle-East, and North, West and Eastern Africa, into agriculture, trade, business, and labor. These diverse pursuits, which complement but also sustain extensive pastoralism, have stimulated the emergence of local elites in regional manifestations of class formation and the embrace of localized variations on globalized culture in lifestyle, fashion, linguistic innovations, the arts (pastoral rap?), tastes, values and philosophy.

This volume on ‘Land Investment & Politics’ represents a creative and energetic look at matters that matter to range-landers as they engage with major projects inspired, engendered and managed by investors who have come for their resources. Its fourteen contributions, each based on long-term community-level research, provide both an overview and concise, in-depth analyses of major sites and situations of development across a huge dryland region that stretches from Somalia and Ethiopia in the Horn of Africa through Kenya to Tanzania. The ambivalence suggested above is revisited through the eyes of indigenous pastoralists and townspeople who ask themselves if these major projects provide them opportunities or offer pivot points of loss. The superb introduction provided in ‘The Politics of Land, Resources & Investment’ brings us up to date on the impact of state planning and the unfolding of major projects and facets of social change in the drylands as towns grow; many herders sedentarize; land is fenced, fragmented and appropriated; and small-scale schemes of irrigation and sand and mineral mining emerge. Four pathways for pastoralists reflect different degrees of access to resources and markets they enjoy: with resources and market access, some people ‘move up’; with resources but low market access, others ‘hang in’; lacking access to resources or markets, some ‘drop out’; and those without resources but able to access markets ‘move out’ of pastoralism. Amidst this global land rush, notwithstanding the continuing local importance of raising livestock, four distinct ways of ‘seeing’ frontiers in space and time are examined in turn: first, like a state, eager to generate wealth that can be ‘captured’; second, like a global investor seeking to tap into repositories of valuable resources while strategically accommodating local political resistance; third, like a post-pastoralist capitalist ‘indigenizing’ the advantages of sedentarization while positioning themselves to meet local demand for livestock and in the process benefitting from heightened inequality; and like an ongoing, struggling pastoralist who falls back on inherent rights of exclusion to exert claims over local land and to struggle for resources accessed through marginal participation in large-scale projects.

This book asks about the wider impacts of major investment projects, both who loses and who wins and how communities are mobilized by the implanting of new projects, some to collaborate, some to resist, and some to do both, as territory is re-shaped and contentious politics emerge. This rich and highly informative book highlights local understanding of investment projects and offers interpretations of how their meaning and importance are framed by diverse agents. Although the power of the state and of international investors may seem dominant, their agents often view events from afar, while those with local interests exercise considerable power, competence and knowledge from below to shape events in their own favour, as these cases illustrate.

Eight of the case studies in fact articulate with the region’s most ambitious integrated development project, LapSSET, or the ‘Lamu Port South Sudan-Ethiopia Transport’ programme, a massive and far-reaching programme of regional infrastructural, energy and social development. For the vast area of northern Kenya (and its borderlands with Sudan, Ethiopia and Somalia), which has long complained that, among other pastoral regions, it has been ignored and overlooked in terms of development initiatives, the LapSSET aims to transform the region by connecting Sudan through the recently discovered Turkana oil fields via a pipeline and road that will extend through Isiolo town to a port being constructed north of the historical coastal island and major tourist site of Lamu, linking the Lamu hinterland with an international airport, a newly established metropolis and a new industrial area. Tracing the path drawn by eight chapters that explore the impact of the LapSSET corridor—from Lamu, through Isiolo, Laikipia and Baringo, to three sites in Turkana County and one in Marsabit County, the book provides a unique vantage point for assessing the processes of investment, resource exploitation and social engagement across the northern Kenyan rangelands. In recent years (Ch. 2), the local Bajuni of Lamu saw their lands grabbed by up-country speculators, but when the new 2010 Constitution mandated the establishment of devolved County-level governance, authorities revoked these corrupt land titles, initiating a re-interpretation of territoriality in terms of exclusion (of Christian non-locals) and inclusion (of local Muslim citizens and political figures). So rather than exemplifying a land grab by outsiders, the ‘high modernist’ LapSSET project is being transformed by struggles over class, gender, generation and ethnicity aimed at making it accord with local realities.

Following the plan to create a ‘gateway city’ in Isiolo (Ch. 3), north-west along the LapSSET corridor, by creating a tourist and an industrial centre, an ‘economy of anticipation’ has emerged that has energized attempts by its many ethnic claimants (Borana, Turkana, Somali, Samburu and Meru) to establish a sense of ‘ownership’ and ‘belonging’. A shift from trust-land held for groups to community land has led locals to carry out land claims and grabs at the periphery of Isiolo as a means of asserting their own ‘inclusion’, as well as in anticipation of rising land values. Further west (Ch. 8), the Kenyan government created the parastatal Geothermal Development Company (GDC) to exploit the energy potential of the geothermal steam fields in the Baringo sector of the Rift Valley, an initiative that began with a major pumping station on the shores of the fresh-water Lake Baringo. To gain local support, the GDC also developed numerous community water points and savings and credit cooperatives to help in recruiting day labourers on construction sites. At the same time, elites have moved to claim land under adjudication, creating anxiety about diminished communal land and stimulating conflict between Pokot and Turkana, a frontier dynamic that predates the ‘extractive frontier’ initiated by the geothermal economy.

Further north (Ch. 4), the discovery of oil in the Turkana South Lokichar Basin in 2012 was followed by attempts by the Anglo-Irish investor, Tullow Oil, and the Vancouver-based Africa Oil corporation to win over local opinion through promising benefits and livelihood opportunities for the local pastoral population. ‘Ethical capitalism’ should involve ‘free and informed consent’, but communities also resort to forms of ‘performance’ to negotiate their access to benefits, through what the authors call ‘inclusion by subversion’. Local youths present themselves for work by day while by night obstructing trucks sneaking oil out of the region, demonstrating an exercise of power from below. Community ‘brokers’ exemplify ‘elite capture’ of potential returns on projects, by both fighting for collective rights and negotiating the terms of their own opportunities. At the same time, in Marsabit, on the eastern shore of Lake Turkana (Ch. 5), where I personally experienced tremendous winds that knocked over my tent on my first trip to the region over 50 years ago, the Lake Turkana Wind Power Project (LTWP) is creating Africa’s largest wind farm through a public-private partnership. Although a key role is defined for Community Liaison Officers (CLO), many are perceived as unjustly favouring their own (Marsabit County) community members in allocating jobs while excluding potential (Samburu) claimants from Samburu County, despite the latter’s views that the region was in fact *enkop ang*, their own land. In effect, given that County boundaries had been drawn during the colonial period to separate Eastern Cushitic and Eastern Nilotic speakers (i.e. Borana, Gabra and Rendille versus Maa-speaking Samburu), the territorial grounding of contemporary identities was being re-negotiated through patronage. Perceived failure to uphold moral obligations led youth to lose faith in elders, who themselves were angry at the chief, resulting in protests and roadblocks aimed at disrupting the project. As large-scale investments threaten to disrupt local systems of territorial ‘belonging’ and social solidarity, they also stimulate resistance from below, which this volume appropriately illustrates.

The Kenyan Government, via the United Nations High Commission on Refugees (UNHCR), has long hosted the globally significant Kakuma refugee camp (Ch. 7) in northern-western Turkana County, which has accommodated hundreds of thousands of refugees fleeing conflict in South Sudan and elsewhere in North-Eastern Africa, creating a mushrooming urban centre in the semi-arid northern rangelands. Responding to local Turkana resentment that refugees were receiving greater benefits than the local population, no matter how poor and deserving, a new ‘alternative to camps’ model for leveraging humanitarian funding to pursue development and economic growth on a regional basis was put into place through the Kalobeyei Integrated Socio-economic Development Programme (KIDSEDP). Though this programme of investment in entrepreneurship and infrastructure is intended to provide aid and economic growth for an intertwined Turkana and refugee population, by creating depots and educational, health, religious and transport services, Turkana still feel they have gained unequal access to the benefits they anticipated would flow from this expanded refugee settlement.

Broadening its scope to other key sites of investment and development in Eastern Africa, the book presents other complementary regional studies that illustrate contexts where states expel landowners in favour of investments, many of which fail in their own terms. Expulsion of villagers to make way for rice cultivation by the Kilombero Plantation Ltd. in Tanzania leads to dispossession, resistance and bankruptcy (without returning to the *status quo*) (Ch. 6). The Gulf states invested in the Berbera Corridor in Somaliland to cement their geo-strategic standing, precipitating a scramble for access to the refurbished port, and thus control of the regional livestock trade, by the clan-based economic networks, which may cause future conflict and insecurity (Ch. 9). Burdened by highland Ethiopian stereotypes of their ‘backwardness’, ethnic groups of the South Omo have seen huge amounts of their land taken by the government and allocated to foreign investors for commercial sugar-cane plantations and conservation schemes as exercises in state-building but resulting in local impoverishment (Ch. 10). Promises by a Swedish investor to produce sugar, ethanol and electricity through an irrigation scheme in Bagamoyo, in eastern Tanzania, with development funding, stripped locals of their land, but delays in implementation and delays in settlement have led to failure without restitution (Ch. 11). Sales of commercial ranches to land-buying companies in Laikipia, using loans from the Agricultural Finance Corporation (AFC), have led shareholders to settle without title deeds on land that is unsuitable for agriculture, in the opportunistic interests of political actors (Ch. 12). A farmer-led irrigation scheme on the Turkwell has been encouraged by the government, as an alternative to the regional livestock economy, which failed as productive investments, while small-scale initiatives have in fact thrived and sustain local livelihoods (Ch. 13). Despite the conflict between the Afars and the Issa Somalis over rights in the Awash Valley, investments in state farms were made, stimulating violence along this ‘investment frontier’ and the emergence of ‘individualistic adaptations’ as some Afar pastoralists have become domesticated capitalists in a setting of increasing inequality and conflict (Ch. 14).

The case studies in this volume spend little time on the motivations of international investors or the economic returns expected from major projects of resource and infrastructural development and in this sense emphasize less what the Introduction called ‘Seeing like a global investor’ than on the perspectives of the state, communities of pastoralists, and the local elites that seek to mobilize local capital. I would propose that two conclusions can be drawn regarding the impact of the major investments being made in Eastern Africa, which are reported here with great depth of knowledge and nuance. Firstly, the expectations have not yet been fulfilled that opening the region to major investments, underwritten by private and public capital, including major development agencies, would bring greater national and local prosperity; but nonetheless, spin-off (or trickle-down) benefits through employment have proven worth fighting for in pastoral regions. Secondly, the predictions that a public-private alliance of state and investor interests would squelch local interests while taking their land may have been too pessimistic, since in every case land holders and their local elites have found means of resisting their exclusion by using on-the-ground pressure tactics that amount to negotiations and in this way have protected their interests while salvaging access to benefits. The conclusion seems to be that, although fine plans can be designed in national and international capitals and agreements signed, those with little voice will in the end have their say. Major projects have taken land, which has fed ongoing disputes, but have not displaced local agrarian economies from the centrally important place they have long occupied, even as those livelihoods have undergone transformation through the growth of towns, the engagement in settlement, and the efflorescence of institutions providing services in health, education, markets and civil society, and class formation. Major investment projects are not rejected locally but demands are made of them by those following local pathways: those ‘moving up’ seek to be brokers for outside interests, those ‘hanging in’ resist displacement and those ‘dropping out’ seek and demand casual employment, while those ‘moving out’ explore access to regular employment. It is remarkable how many major projects either have failed or have fallen short, leaving the threats of very large-scale land appropriations unrealized as local persistence tends to win out. The cases reviewed here hint at a strategy for the future, in authentic partnerships within the localities where resources can be exploited, building on local awareness and knowledge to create well-founded projects designed to bring more broadly disseminated benefits, for investors, states and communities, and for those who await the resources being developed in renewable energy, food and infrastructure.

## Data Availability

Not applicable.

## Abbreviations

AFC: Agricultural Finance Corporation (Kenya)
GDC: Geothermal Development Company (Kenya Parastatal)
KIDSEDP: Kalobeyei Integrated Socio-economic Development Programme
LapSSET: Lamu Port South Sudan-Ethiopia Transport Programme
LTWP: Lake Turkana Wind Power Project
NGOs: Non-governmental organizations
UNHCR: United Nations High Commission on Refugees

